# Factors related to treatment adherence behaviors among old-age hemodialysis patients in Hamadan, Iran: the application of the extended theory of planned behavior during Covid-19 pandemic

**DOI:** 10.1186/s12882-022-02694-x

**Published:** 2022-02-07

**Authors:** Vida Sheikh, Majid Barati, Salman Khazaei, Hanieh Jormand

**Affiliations:** 1 Clinical Research Development Unit of Shahid Beheshti Hospital, Hamadan University of Medical Sciences, Hamadan, Iran; 2 Department of Public Health, School of Health and Autism Spectrum Disorders Research Center, Hamadan University of Medical Sciences, Hamadan, Iran; 3 Department of Epidemiology, School of Health and Research Center for Health Sciences, Hamadan University of Medical Sciences, Hamadan, Iran; 4 Autism Spectrum Disorders Research Center, Hamadan University of Medical Sciences, Hamadan, Iran

**Keywords:** Adherence, Chronic Kidney Disease, Aging, Theory of Planned Behavior, COVID-19

## Abstract

**Purpose:**

This study aimed to identify the factors related to treatment adherence behaviors among old-age hemodialysis patients in Hamadan based on the Extended Theory of Planned Behavior (ETPB) during the covid-19 pandemic.

**Methods:**

This cross-sectional study was conducted from January to March 2021 in Hamadan, Iran. 191 hemodialysis patients were recruited who were referred to hemodialysis centers via the census method. Data were collected by a questionnaire containing items on socio-demographic information, End-Stage Renal Disease Adherence (ESRD-Adherence) Questionnaire, and ETPB constructs scale. Data analysis was performed using descriptive statistics and structural equation modeling.

**Results:**

The mean (SD) age of participants was 62.49 (10.66). Also, the mean (SD) hemodialysis vintage/Month of them was 36.56 (43.34). Moreover, Treatment Adherence Behaviors are associated with education level, sex, and marital status (*p* < 0.001). Besides, Perceive Behavior Control (β = 0.414, t-value = 3.810) associated with intention. Also, intention (β = 0.158, t-value = 1.976) was associated to adherence behaviors. No significant associations were observed between positive attitudes, subjective norms, a perceived threat with intention, and adherence behaviors. The model explained about 54% of the variance of adherence behaviors. Finally, the goodness of fit index of 0.78, indicating the model good fit.

**Conclusion:**

The present study demonstrates that some of the ETPB constructs such as perceived behavior control and intention are useful to improve adherence among the oldest hemodialysis patients. Also. This framework is revealed alongside the theory of planned behavior (TPB) applicable in the treatment adherence of old-age hemodialysis patients.

## Introduction

Chronic kidney failure is one of the most common non-communicable diseases that occurs during the progressive process of decreased nephron function. The end stage of the disease (end-stage renal disease) leads to disability in the patient [1].

According to the World Health Organization, treatment adherence is the correspondence level of a person receiving medication, following a prescriptive diet, or implementing lifestyle changes due to healthcare providers’ recommendations [2]. Adherence to hemodialysis (HD) for patients with end-stage renal disease is essential and leads to lifestyle changes such as the need to regularly go to a dialysis center, consistently take prescribed medications, and extensively modify their diets [3]. Also, successful treatment of HD patients depends on maximum patient adherence to treatment. Evidence demonstrated that four behaviors could result in the decreased quality of life, increased morbidity, healthcare costs, and mortality in these patients [4]. However, management of renal replacement therapy includes behaviors such as HD attendance, medications, fluid restrictions, and diet prescription [4, 5]. The result of the study indicated that HD patients needed complex care to manage this chronic disease [6]. In contrast, a study matched with the Iranian context showed that it was essential to identify factors effective for treatment adherence in HD patients [7].

### The treatment adherence concept

Treatment adherence plays a critical role in decreasing the economic burden of disease for families and society [8, 9]. Patient non-adherence in the literature can be considered a patient’s “incomplete implementation of instructions” issued by a provider [8, 10]. The results of studies proved average patient non-adherence rates of 24.8% across physician-recommended treatments types, with some non-adherence rates approaching 40% [8, 11, 12]. The type of treatment implemented significantly impacts treatment adherence and non-adherence, with treatment regimens such as lifestyle or behavior changes presenting rates of non-adherence of up to 70% [8, 13].

### The conceptual framework

The model-based approach assists in promoting or changing behavior by understanding adherence to health behavior. This approach includes the Health Belief Model, Theory of Planned Behavior (TPB), Theory of Reasoned Action, and other theories or models [14]. TPB is a social-cognitive approach that provides a useful framework for predicting and understanding health-related behaviors [15, 16]. According to this theory, intention is the primary determinant of behavior. The individual’s intention is influenced by the three factors of attitude, subjective norms, and perceived behavioral control (PBC) [17, 18]. Evidence demonstrated several important factors that could predict and affect adherence behavior in patients, such as attitude, subjective norms, PBC, and intention [19, 20]. On the other hand, a perceived threat to treatment adherence can play a decisive role in adherence behaviors in HD patients [21].

**Fig. 1:**
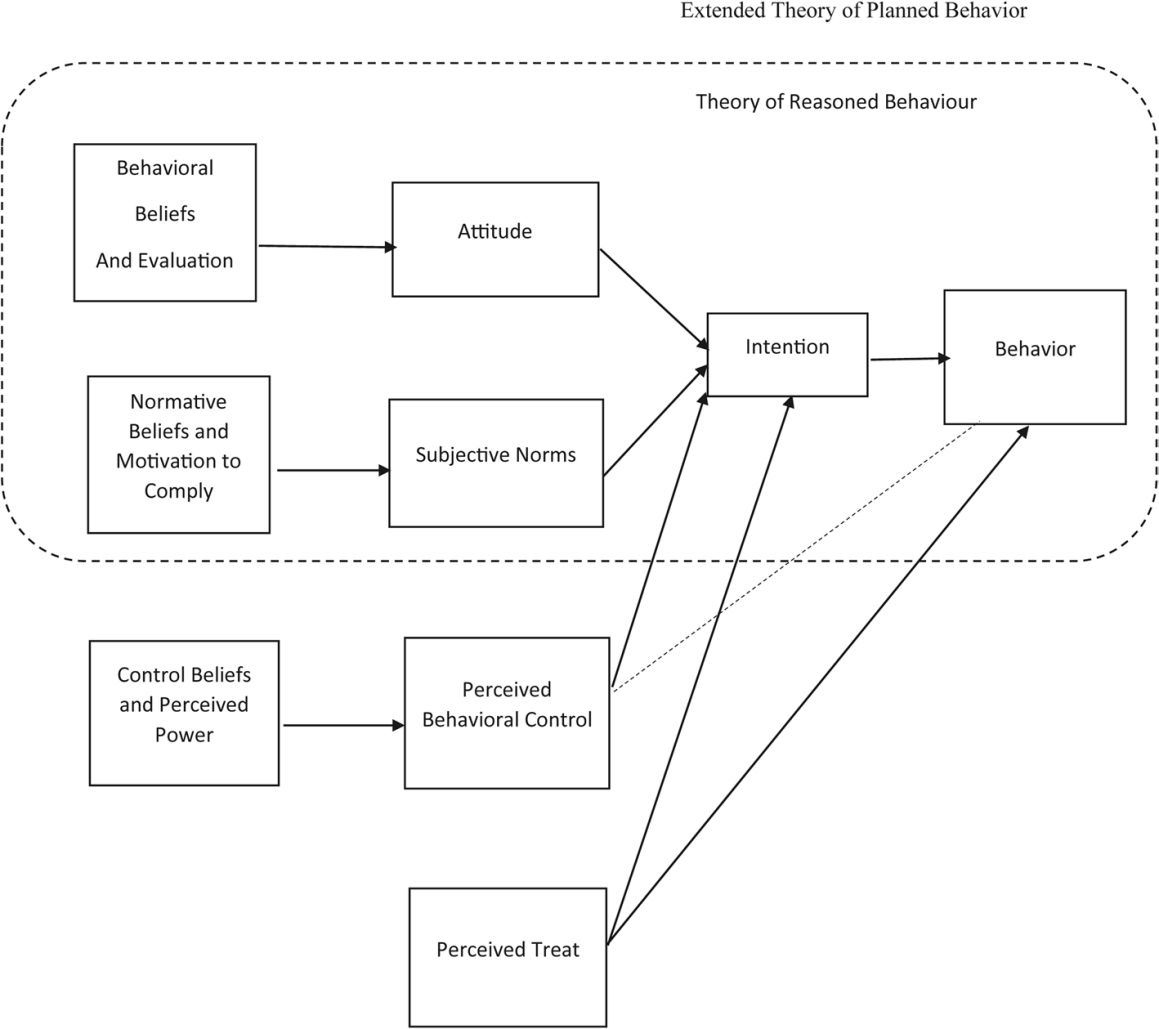
The framework of the Extended Theory of Planned Behavior

Therefore, the Extended Theory of Planned Behavior (ETPB) was used in this study to identify factors affecting treatment adherence behaviors. In this framework, perceived threat includes perceived susceptibility, and perceived severity is added to TPB (Fig. 1).

Also, since December 2019, coronavirus (COVID-19) has become a global pandemic, and results of clinical studies showed that the risk of COVID-19 infection hospitalized was more than 10% in dialysis patients and 6.4% in in-hospital medical staff [22]. Additionally, concerns increased about non-adherence behaviors in HD patients, especially in the COVID-19 pandemic [23, 24]. Thus, the present study aimed to identify factors related to treatment adherence behaviors among older HD patients in Hamadan, Iran, based on ETPB.

## Methods

### Design and participants

This cross-sectional study was conducted from January to March 2021 in Hamadan, Iran. Hamadan Province, with an area of 19,493 square kilometers in extent, is located in western Iran. We used patients’ information from two hospitals with dialysis wards, including Besat and Shahid-Beheshti in Hamadan city. A total of 191 HD patients were recruited and referred to HD centers via the census method. Participants were selected based on the following inclusion criteria: being diagnosed with end-stage renal disease and treated with HD for at least three months; receiving HD for three to four hours per session, three times per week; ability to complete the survey and willingness to participate in the study; and ability to give informed consent. Patients on transient HD due to acute renal failure were excluded.

### Data collection

All methods were performed following relevant guidelines and regulations. Verbal informed consent was obtained from all the patients; they were informed about the confidentiality of the information and the project’s purpose, and they were enrolled in the study if they liked. The Ethics Committee approved the study with the consent process at the Hamadan University of Medical Sciences.

### Measurements

The data collection tool used in the study consisted of three parts completed by the participants through the self-reporting method. The first part included demographic information such as age, education level, economic status, marital status, number of families, and COVID-19 morbidity in the participants or their families.

The second section included the 46-item ESRD-Adherence Questionnaire (ESRD-AQ) designed by Kim et al. [4] that assesses four behaviors: HD attendance, medications, fluid restrictions, and diet prescription.

The ETPB constructs were assessed using the ETPB scale for treatment adherence behavior designed and regulated by the researcher through the study of books, papers, and literature [25, 26]. It was compiled of 43 items and nine subscales as follows: behavior beliefs (seven items, e.g., “Attending HD sessions makes me feel happy, relaxed and at peace”), outcome evaluation (seven items, e.g., “I need to take prescription drugs on time and correctly.”), normative beliefs (four items, e.g., “My children or my family thinks that I should take my medication on time and correctly.”), motivation to comply (four items, e.g., “For me, the opinion of my children and family that I should take my medication on time and correctly is important.”), control beliefs (six items, e.g., “I regularly attend recommended dialysis sessions, even if the HD session is long.”), perceive power (five items, e.g., “I do not attend an HD session because of travel problems.”), perceive susceptibility (three items, e.g.,“ Compared to other people my age, I am more likely to have coronavirus.”), perceive severity (six items, e.g., “If I get Covid-19, my life will be shortened..”), intention (three items, e.g., “I plan to attend regular HD sessions.”), and the treatment adherence behaviors (46 items, e.g., “During the last month, how many dialysis treatments did you miss completely?”). These items were assessed using a 5-point Likert scale from 1 (strongly disagree) to 5 (strongly agree). The score of each subscale was obtained by the average computed as the sum of items of it [4]. It is essential to mention that based on a previous study [4], the patients were categorized in two levels, “adherence” and “non-adherence,” based on biological and biochemical markers of clinical outcomes such as degree of weight gain (increase in weight in kg after a weekend), serum potassium levels (average of last three measures) in meq/l, serum phosphate levels (average of last three measures) in mg/dl, and serum albumin (average of last three measures) in mg/dl [27–29].

The content validity ratio (CVR) and the content validity index (CVI) were used to determine content validity. The results showed high overall CVI and CVR of the ETPB scale. The means scores for CVI and CVR were 0.92 and 0.80, respectively. The CFA results confirmed goodness of fit the ETPB sub-constructs. The discriminant validity was verified using the Fornel-Larcker and Average Variance Extracted (AVE) methods. The composite reliability (CR) range and Cronbach’s alpha range [30]. The composite reliability ranged from 0.86 to 0.98, and Cronbach’s alpha ranged from 0.76 to 0.98.

## Results

### Sample characteristics

In this present study, with 191 participants, 47.8% were female (*n* = 93) and, with a mean age of participants was 62.49 ± 10.66. Also, the mean HD vintage (Month) of them was 36.56 ± 43.34.

According to the findings, Treatment Adherence Behaviors associated with education level, sex, and marital status as treatment adherence behaviors in a patient with secondary education level with OR = 0.17 (95% CI 0.04, 0.75) and, in female patients with OR = 0.29 (95% CI 0.09, 0.94) (*p* < 0.05), while, in married patients with OR = 7.70 (95% CI 1.89, 31.44) (*p* < 0.001) were calculated. Other demographic data were presented in Table 1.

**Table 1:** Association between treatment adherence behaviors with demographic variables of participants (*n* = 191)

Variables	n (%) *n* = 191	Non-Adherence (*n* = 17)	Adherence (*n* = 174)	OR(95%CI)	*P*. Value
**Age**					
45–59 (middle-age)	80(41.9)	9(52.9)	71(40.8)	1(Reference)	0.148
60–74 (young-old)	79(41.4)	8(47.1)	71(40.8)	1.13(0.41–3.08)	
75–90 (old-old)	32(16.8)	0(0)	32(18.4)	0(0)	
**Sex**					
Male	98(51.3)	13(76.5)	85(48.9)	1(Reference)	0.030^*^
Female	93(47.8)	4(23.5)	89(51.1)	**0.29(0.09–0.94)**	
**Marital status**					
Single	15 (7.9)	4(23.5)	11(6.3)	1(Reference)	0.004^**^
Married	133(69.6)	6(35.3)	127(73.0)	**7.70(1.89–33.44)**	
Widow/Divorce/Partner Death	43(22.5)	7(41.2)	36(20.7)	1.87(0.46–7.60)	
**Educational status**					
Illiterate	72(37.7)	3(17.6)	69(39.7)	1(Reference)	0.020^*^
Elementary	41 (21.5)	2(11.8)	39(22.4)	0.85(0.14–5.30)	
Secondary	24 (12.6)	5(29.4)	19(10.9)	**0.17(0.04–0.75)**	
Diploma	25 (13.1)	2(11.8)	23(13.2)	0.50(0.08–3.18)	
University	29 (15.2)	5(29.4)	24(13.8)	0.21(0.05–0.94)	
**Job**					
Employee	16(8.4)	3(17.6)	13(7.5)	1(Reference)	0.327
Retire	38 (19.9)	4(23.5)	34(19.5)	1.96(0.39–9.99)	
Labor	7 (3.7)	1(5.9)	6(3.4)	1.39(0.12–16.23)	
No Job	26 (13.6)	1(5.9)	22(12.6)	5.08(0.48–54.03)	
Free Job	23 (12.0)	4(23.5)	22(12.6)	1.27(0.25–6.59)	
Housewife	81 (42.4)	4(23.5)	77(44.3)	4.44(0.89–22.18)	
**Number of Family**					
> 3	94(49.2)	12(70.6)	82(47.1)	1(Reference)	0.181
3–5	56 (29.3)	3(17.6)	53(30.5)	2.59(0.70–9.60)	
> 5	41 (21.5)	2(11.8)	39(22.4)	2.85(061–13.37)	
**Economic status**					
Poor	87(45.5)	9(52.9)	78(44.8)	1(Reference)	0.544
Medium	94 (49.2)	8(47.1)	86(49.4)	1.24(0.46–3.37)	
Good	10 (5.2)	0(0)	10(5.7)	0(0)	
**COVID-19 Morbidity in yourself or family**					
Yes	60(31.4)	7(41.2)	53(30.5)	1(Reference)	0.364
No	131 (68.6)	10(58.8)	121(69.5)	1.35(0.73–2.49)	

Bold font indicates *P-*value is significantly different (*p* < 0.05)

### The ETPB constructs about treatment adherence behaviors

Table 2 shows the means and standard deviations of the ETPB constructs. Participants rated perceived behavioral (PBC) (84%), perceived threat (84%), Intention (79%), positive attitude (75%), subjective norms (70%), which were the highest percentage of the mean from the maximum obtainable score. The mean of constructs showed that all constructs were considered desirable (Table 2).

**Table 2:** The mean ± SD and correlation between the ETPB constructs matrix in hemodialysis patients

**Constructs**	Mean ± SD	**Percentage **^**a**^	**Rang of Score**	**Positive Attitude**	**Subjective Norms**	**Perceive behavior control**	**Perceive threat**	**Intention**	**Treatment** **Adherence Behaviors**
Behavior Beliefs	27.57 ± 6.76	73.46	7–35	1	0.273^c^	0.128	0.105	0.158^b^	0.202^c^
Outcome Evaluation	27.28 ± 7.05	72.43	7–35						
**Positive Attitude**	55.84 ± 13.55	74.71	14–70						
Normative Beliefs	15.49 ± 4.41	69.93	5–20	0.273^c^	1	0.353^c^	0.705^c^	0.318^c^	0.717^c^
Motivation to Comply	15.51 ± 4.44	70.07	5–20						
**Subjective Norms**	31.00 ± 8.72	70	10–40						
Control Beliefs	26.72 ± 3.20	86.88	5–30	0.128	0.353^c^	1	0.408^c^	0.480^c^	0.258^c^
Perceive Power	20.90 ± 3.83	79.5	5–25						
**Perceive Behavior Control**	47.62 ± 6.27	83.6	10–55						
Perceive Susceptibility	13.04 ± 3.03	83.67	3–15	0.105	0.705^c^	0.408^c^	1	0.340^c^	0.718^c^
Perceive Severity	17.38 ± 3.85	83.63	4–20						
**Perceive Threat**	30.42 ± 6.76	83.64	7–35						
**Intention**	12.53 ± 2.36	79.42	3–15	0.158^b^	0.318^c^	0.480^c^	0.340^c^	1	0.381^c^
HD Treatment	564.01 ± 84.94	94.0	0–600	0.202^c^	0.717^c^	0.258^c^	0.718^c^	0.381^c^	1
Medication	188.48 ± 38.02	94.24	0–200						
Fluid Restriction	164.92 ± 61.13	82.46	0–200						
Dietary Restriction	168.85 ± 50.37	84.43	0–200						
**Treatment Adherence Behaviors**	1086.26 ± 163.99	90.52	0–1200						

^a^ percentage of the mean from the maximum obtainable score. ^b^Significant at the 0.05 level. ^c^Significant at the 0.01 level

### Associations between the ETPB constructs

Although, based on results there was a positive direct positive association between intention with attitude (*p* = 0.05), PBC, perceived threat (*p* < 0.01). While there was a positive direct positive correlation between treatment adherence behaviors with attitude, PBC, intention (*p* < 0.01).

However, perceive a threat (*r* = 0.33) and perceive behavior control (*r* = 0.27) were more correlated with intention among other extended theories of planned behavior constructs.

Though, intention (*r* = 0.33) and PBC (*r* = 0.38) were more correlated with treatment adherence behaviors among other ETPB constructs (Table 2).

### The structural modeling analysis

**Fig. 2:**
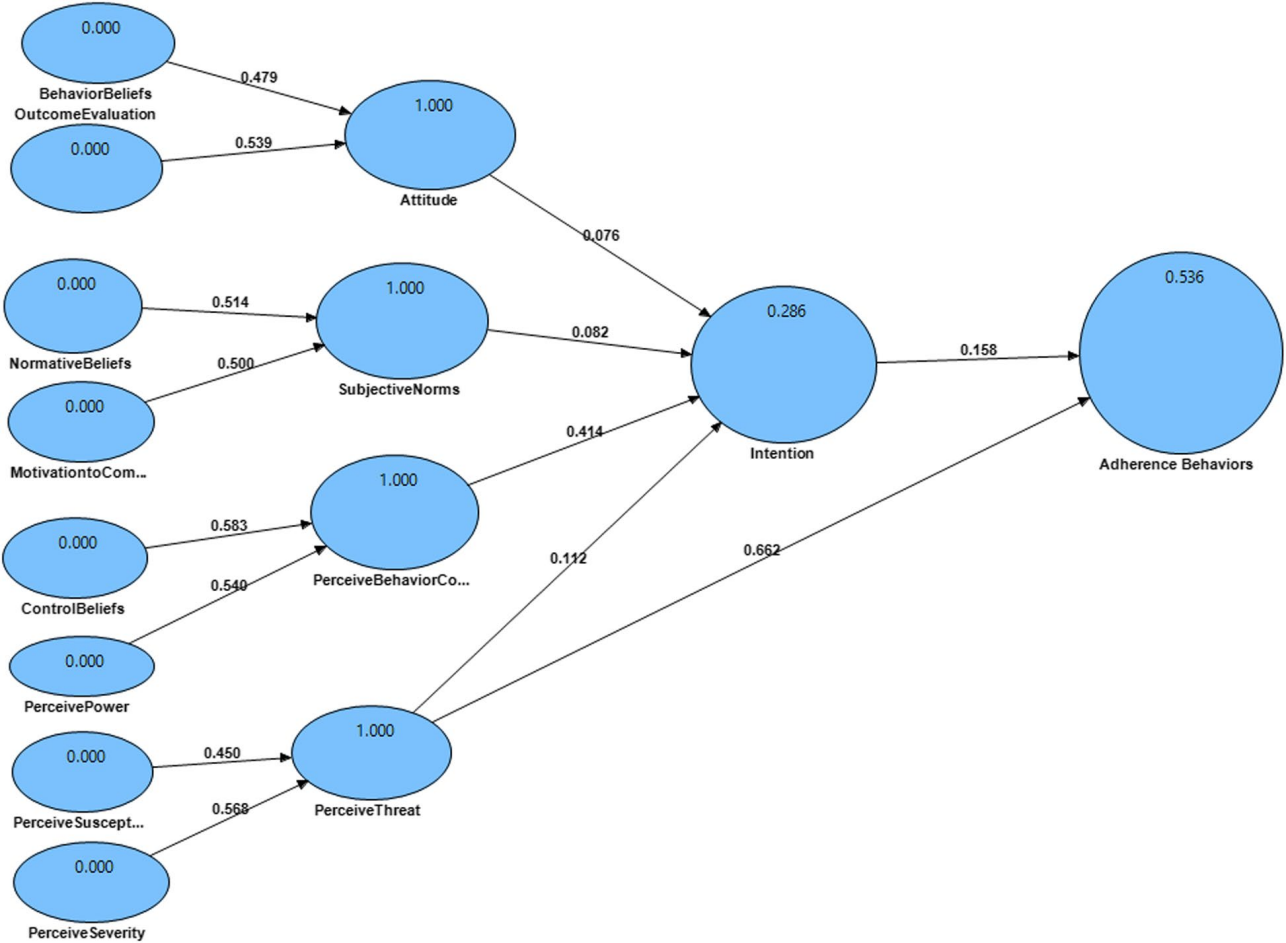
Structural Equation Modeling of the Extended Theory of Planned Behaviors

Table 3, Fig. 2 presented the results of analyzing, the model 54% of the variance of adherence behaviors as the dependent variable (R square = 0.536). The R square for intention is 0.286 that suggests 29% of the variance of intention can be explained by PBC.

**Table 3:** Path Analysis of the ETPB Model (*n* = 191)

Dependent Variable	Independent Variable	Path Confidence	t-value	The significance level	R-Square
Intention	**Positive Attitude** **Subjective Norms** **Perceive Behavior Control** **Perceive Threat**	0.076 0.082 0.414 0112	0.743 0.705 3.810 0.795	0.302 0.310 **0.001**^**b**^ 0.290	0.286
Adherence Behaviors	**Intention**	0.158	1.976	**0.05**^**a**^	0.536

^a^ Significant at the 0.05 level. ^b^ Significant at the 0.01 level

The PBC (β = 0.414, t-value = 3.810) associated with intention. Also, intention (β = 0.158, t-value = 1.976) was associated to adherence behaviors. No significant associations were observed between positive attitudes, subjective norms, perceived threat with Intention, and adherence behaviors (Table 3).

The model’s predictive power was tested by calculating Q2 indexes of intension (Q2 = 0.672) and adherence behaviors (Q2 = 0.557), exceeding the recommended threshold value (Q2 > 0), demonstrating an adequate predictive value of the model. Finally, GoF = 0.78, indicating the model good fit (Table 4).

**Table 4:** Goodness of fit values for models

Variable	AVE > 0.4	CR > 0.7	*R*^2^	Communality > 0	Redundancy
Positive Attitude	0.780	0.980	1	0.780	0.554
Subjective Norms	0.797	0.980	1	0.797	0.587
Perceive Behavior Control	0.469	0.902	1	0.469	0.329
Perceive Threat	0.778	0.961	1	0.778	0.621
Intention	0.672	0.859	0.536	0.672	0.013
Adherence Behaviors	1	1	0.285	1	0.098

*AVE* Average Variance Extracted, *CR* Composite Reliability, *R*^*2*^ = R Squares

## Discussion

The current study utilized the ETPB framework to identify factors affecting treatment adherence behaviors among HD patients. It was shown that TPB could predict and improve adherence behaviors of chronic kidney disease patients, which is in line with Chironda et al.’s results [14].

According to the findings, treatment adherence behaviors are associated with the education level. Recently, similar to our results, Kim has reported that with increasing the education level, treatment adherence behaviors are improved [31]. We can mention that patient noncompliance is attributed to personal qualities of patients, such as forgetfulness, lack of willpower or discipline, or low level of education [32]. Although, patients need to clearly and appropriately understand health information related to their specific illness or disease [33, 34]. This understanding may be essential to helping patients generate beliefs and attitudes and improve adherence behaviors [34]. Thus, when patients are adequately informed and understand clearly what they are asked to do, they are better able to share in decisions that affect their health and are thus more adherent to treatments [33, 35]. On the other hand, it seems that mastery experiences and social roles in high educational individuals caused to increase self-efficacy and PBC, which other results of several studies proved to have higher PBC lead to the formation of a behavioral intention, and performance of behavior [16, 18, 36, 37]. Therefore, it is recommended to design and plan educational interventions to improve PBC and intention for all HD patients, especially low-educated individuals.

Based on the present study, treatment adherence behaviors associated with sex were better in females than in males. Consequently, gender should also affect treatment adherence behaviors [38]. However, a relationship between gender and adherence has not been consistently shown in the literature. Several studies have found women to be more non-adherent than men [39–41]. Others have reported that women tend to follow their prescriptions better than men [42, 43]. Nevertheless another study mentioned no difference between male and female regarding drug adherence [44]. These conflicting results may be due to different conditions and treatments compared to the present study. Thus, it is recommended to plan educational intervention to improve treatment adherence behaviors for all HD patients.

Based on findings, treatment adherence behaviors are associated with marital status. In line with this result, Turan et al.’s study showed that social supports affected patients’ adherence to the treatment [45]. Generally, evidence was emphasized that family members or peer supports may promote better adherence in some patient groups [46]. So, it is recommended to plan educational intervention to improve treatment adherence behaviors for singles, widows or widowers, the divorced, or partners death HD patients.

Although all ETPB constructs were considered desirable. In line with this finding, Beerappa et al. reported a fair to good adherence level for fluid and dietary restrictions in patients [47].

Based on results, PBC is associated with intention and among the constructs of ETPB, PBC plays a significant role in explaining the variance of the intention of treatment adherence behaviors among HD patients, which is similar to Biddle’s study, showing that having higher PBC was a predictor of improved self‐care [39]. Based on TBP, PBC over behavior performance can explain great variance in intentions and actions [48].

This study found that intention was associated with adherence behaviors, as mentioned in previous studies (Husebø et al.) which [49] intention to engage in the health‐changing behavior and PBC demonstrated significant association with adherence behaviors. Moreover, Finchman et al. revealed possible decreased medical complications and mortality due to PBC and intention to improve adherence to the dietary and fluid restrictions among HD patients [50].

This study has several limitations. First, this was a cross-sectional study that enrolled a small number of patients. Thus, it is recommended to identify additional factors in future research. Second, future research should analyze data from a larger sample of participants. In contrast, the potential for recall and interviewer biases may be included, and a longitudinal study design could be useful for managing bias. The findings of this study might not be generalized to all populations of older people. Therefore, future research can investigate predictors of treatment adherence behaviors from a more ecological approach by examining TPB constructs complemented by wider levels of individual and social factors.

## Conclusion

The present study demonstrated that some of the ETPB constructs, such as PBC and intention, were useful to improve adherence among older HD patients. Also. the framework revealed alongside TPB is applicable in the treatment adherence of older HD patients. Thus, future studies are suggested to pay more attention to increasing PBC and intention in designing and implementing educational programs. Interventions can also heighten perceived behavioral control to increase treatment adherence behaviors among HD patients.

## Data Availability

The datasets used and/or analyzed during the current study are available from the corresponding author on reasonable request.

## Abbreviations

ETPB: The Extended Theory of Planned Behavior
TPB: The theory of planned behavior
HD: Hemodialysis
CVR: Content validity ratio
CVI: Content validity index
PBC: Perceived behavioral control
